# The learning space—interpersonal interactions between nursing students, patients, and supervisors at developing and learning care units

**DOI:** 10.1080/17482631.2017.1368337

**Published:** 2017-09-18

**Authors:** Hanna Holst, Lise-Lotte Ozolins, David Brunt, Ulrica Hörberg

**Affiliations:** ^a^ Department of Health and Caring Sciences, Linnaeus University, Växjö, Sweden

**Keywords:** Clinical practice, learning in pairs, patient perspective, lifeworld-led support, caring science, reflective lifeworld research

## Abstract

**Purpose:** Previous research shows that the learning space is significant for students’ learning in pairs in clinical practice but does not explain the meaning of the phenomenon. The aim of this study is thus to explain and understand the learning space that occurs in the interaction between the patients, the pairs of nursing students, and the supervisors on a developing and learning care unit in Sweden. **Method:** The study has been carried out with a Reflective Lifeworld Research (RLR) approach founded on hermeneutics. A total of 39 informants, consisting of 16 patients, five pairs of students (10 students), and 13 supervisors, were observed and interviewed. **Results:** The results reveal that an interpersonal linkage between the patients, the students, and the supervisors is created within the learning space. A learning space, based on respect towards each other, creates the prerequisite for beneficial and supportive interactions that contribute to a deeper relationship. **Conclusion:** The phenomenon is complex due to its expandable nature and due to the fact that the learning space cannot be isolated from the surrounding environment. In order to exploit the potential of the learning space it is of importance to understand and consider the learning space as a whole.

## Introduction

Two of the major issues in nursing education concern the integration of theory in practice and the creation of collaboration between the clinic and academia (Edgecombe & Bowden, 2014). A number of models have been developed to support nursing students in these aspects. These models include a focus on the context—DEUs (dedicated education unit) and DLCUs (developing and learning care unit) (Ekebergh, 2007), on students learning together—peer learning (Edgecombe, Wotton, Gonda, & Mason, 1999; Nygren & Carlson, 2017; Stenberg & Carlson, 2015) and on learning in pairs (Hörberg et al., 2014). The aim of all these models is to support students’ learning by supervision in structured learning environments. The structured learning environment in this study is a DLCU and the focus is on the learning space created by interaction between pairs of nursing students, patients, and supervisors.

Nursing students experience the learning environment at a DEU as challenging because of the expectations of intertwining theory in practice (Edgecombe & Bowden, 2009; Eskilsson, Hörberg, Ekebergh, & Carlsson, 2014). At the same time, the nursing students experience a potential for developing when learning at DEUs (Grealish & Ranse, 2009; Moscato, Miller, Logsdon, Weinberg, & Chorpenning, 2007; Ranse & Grealish, 2007; Wotton & Gonda, 2004) both in reaching the goal for their course and in developing their nursing skills in the encounters with the patients (Aston & Molassiotis, 2003; Edgecombe & Bowden, 2009; Stone, Cooper, & Cant, 2013). Patients cared for at a specific DEU with a lifeworld perspective experience genuine care by the nursing students, which is experienced as a contact with them that is characterized by closeness, thoroughness, accessibility, acknowledgement, and sensitivity (Eskilsson, Carlsson, Ekebergh, & Hörberg, 2015). Further, patients also experience feelings of safety when being invited to participate in the caring actions by the nursing students as they are supported by the supervisors (ibid.). The latter experience the learning environment as both challenging and developing. It is a challenge for supervisors to be involved in both caring and learning through supervision at a DEU with a lifeworld perspective, but at the same time it is an opportunity for personal development, where building a sense of togetherness with the nursing students is an important part (Eskilsson, Hörberg, Ekebergh, Lindberg, & Carlsson, 2015). An essential element of supervising in lifeworld-led learning is supporting by listening, seeing, and feeling in order to be aware of the nursing students’ needs for support (Ekebergh, 2009).

Furthermore, the encounters between nursing students and their supervisor have been highlighted with a focus on the relationship between the two. Supervisors describe seeing the nursing students as a whole person or a unique individual, which creates a sense of trust as a base for their relationship (Pierson, 1999; Ross, Head, King, Perry, & Smith, 2014). Patients also consider themselves as included in a social and professional interaction when the nursing students and the supervisors are friendly towards one another based on good cooperation (Stockhausen, 2009). Similarly, the nursing students experience the relationship with the patients as important for their personal and professional growth and highlight the encounters as the basis for their learning (Manninen, Henriksson, Scheja, & Silén, 2012).

Nursing students also describe the relationships with other student colleagues as playing an important role in their learning in clinical practice. Peer learning and learning in pairs reveal positive experiences when the students support each other in developing knowledge and in their new role as beginner practitioners (Aston & Molassiotis, 2003; Edgecombe & Bowden, 2009; Holst & Hörberg, 2013, 2012; Stenberg & Carlson, 2015; Stone et al., 2013). The relationship between nursing students is described as a forum for their feelings of safety and reduced anxiety when learning together. The safety is based on the nursing students’ experiences of being able to ask questions, reflect, and face challenges together. Students also experience being responsible for their own knowledge when taking turns in teaching each other, but they also experience a sense of competition regarding the attention from their supervisor (Holst & Hörberg, 2012; Stenberg & Carlson, 2015).

Previous research reveals similarities between the concept of peer learning and learning in pairs (Holst & Hörberg, 2012, 2013; Nygren & Carlson, 2017; Stenberg & Carlson, 2015). There is, however, a considerable difference between these two ways of supporting students’ learning, namely the concept of learning in pairs is based on a lifeworld perspective where the pairs are seen as a unit. To our knowledge, only a few studies (Holst & Hörberg, 2012, 2013) focus on the opportunity for student learning in pairs from a lifeworld perspective in clinical practice. Learning in pairs of students can be described as collaboration between the students within the pair throughout the whole period of clinical practice. The supervisor supports each individual nursing student and the students as a pair based on a lifeworld-led learning approach, which focuses on each student’s experiences and knowledge (Holst, Ozolins, Brunt, & Hörberg, 2017). However, previous research concerning nursing students’ learning in pairs also reveals the importance of interpersonal meetings in order to support students’ learning in clinical practice. These interpersonal meetings are described as creating a learning space between the students and the supervisors with a basis in the patients’ care. Furthermore, the learning space is shown to provide support for nursing students’ learning by stimulating their independence and in the sharing of knowledge with each other (Holst & Hörberg, 2012, 2013; Holst et al., 2017). Further research is required in order to be able to understand the potential of the phenomenon “the learning space” within DLCUs, in supporting students’ learning. The aim of this study is thus to explain and understand the learning space that occurs in the interaction between patient, pairs of nursing students, and supervisors.

## Method

### Design

This hermeneutic study is based on reflective lifeworld research approach (Dahlberg, Dahlberg and Nyström, 2008). The concept of the lifeworld is based on Husserl’s (1970/1936) theory of understanding and describing the lived experience of a phenomenon. In order to understand the lived experience, existential interpretations can suggest how a person is able to understand his/her situation in the world (Ödman, 2007). The lifeworld can be reached through an open attitude, which entails sensitivity towards the things being investigated (Dahlberg et al. 2008). According to Gadamer (1960/2004) our pre-understanding is a prerequisite for our understanding, but in order to adopt a researching approach a critical attitude towards the pre-understanding must be held. This could be reached by an open approach, which entails becoming aware that our pre-understanding is influenced by the culture and history we live in. Further, an open approach is required throughout the analysis in order to reach understanding of the “otherness” of the research phenomena (Gadamer, 1960/2004). The focus within this hermeneutic approach is to understand and explain the latent meaning in data in order to reach new understanding (Ricoeur, 1976).

### Clinical settings

The study was conducted in a learning environment based on the DLCU model (Hörberg et al., 2014) in a general hospital. Three units with different specializations are included in this study, medicine, orthopaedic, and surgery. The basic concept of the model is to, with a reflective approach, bridge the gap between theory and praxis by supporting nursing students’ learning when supervised in pairs. The team of supervisors consists of a head supervisor (nurse) who is responsible for the nursing students’ clinical practice, a base supervisor (nurse) who supervises nursing students in the bedside area, and a lecturer (from nursing education) who contributes with the theoretical caring science perspective. A pair of students in this model consists of one student from the second year and one student from the final third year. The students collaborate during a 5-week period of clinical practice.

### Participants

A total of 39 informants consisting of five pairs of nursing students (10 students), 16 patients (being cared for by pairs of students) and 13 supervisors (three head supervisors and 10 base supervisors) participated in the study. A strategic sample of pairs of students representing a variation in age, gender, and experience in the bedside area was sought and attained. A sample of patients and supervisors was selected from those nursing students who participated in the study. Inclusion criteria were: patients who can express their experiences and whose status would not be compromised by participating, nursing students being supervised in pairs, and supervisors with experience of supervising pairs of nursing students (termed student(s) in the following text).

### Data collection

The data consisted of observations and interviews and were collected by the first author during two periods of a 5-week clinical practice. Each pair of students was observed and interviewed (both individually and in pairs) on three occasions during their clinical practice (see Table I). The patients and the supervisors who interacted with the students during the observations were also included in the study and interviewed individually. Data were first collected in the form of observations by following a pair of students and their encounters with the included patient and supervisors. The observations lasted for 4 to 5 hours for a total of 15 days. The observer remained in the background, dressed in nursing uniform, available for answering questions, but not participating actively in the care of the patient. The focus of the observations was the interaction between the pairs of students, patients, and supervisors. Field notes were written down after each observation in a separate room and were intended to provide a detailed description of the understanding of the interactions. Each observation was followed by an interview with each of the participants. The recorded interviews, which lasted for approximately 10 minutes with the patients and approximately 20 minutes with the students and the supervisors, were held in a separate room at the unit. The purpose of the interviews was to capture the experiences of the participants in the observed encounters with a focus on the phenomenon “learning space” between patient, students, and supervisor. Examples of questions that the participants were asked are:

- To the patient: how do you experience the encounter with pairs of students when they are caring for you?
- To the students: could you describe the encounter you had together with the patient, student colleague, and supervisor?
- To the supervisor: how do you experience the encounter between you, the students, and the patient?

**Table I.** Data collection.

	Students	Patients	Supervisors
Participants (total no. = 39)	10 (5 pairs)	16	13
Interviews (total no. = 51)	5 in pairs; 15 individual	16	15

In order to capture a deeper understanding of the experiences of the phenomenon in focus follow-up questions such as “Could you describe the feelings you had during the encounter?” or “Would you like to give me an example?” were asked. During the interviews a reflective dialogue was pursued in order to enable the participants to describe their experiences of the phenomenon.

### Data analysis

The analysis process was carried out in accordance with the RLR approach founded on hermeneutic traditions (Dahlberg, Dahlberg & Nyström, 2008). All the interviews were transcribed verbatim and were read several times to get to know the material. Meanings of the phenomenon “learning space” were searched for and highlighted in the interviews. Questions such as “Is this a description of the learning space?” were asked of the text. The highlighted meanings were sorted and grouped in to themes according to their similarities and differences. The meanings within each theme were first interpreted on a level close to the data with an open and reflective mind in order to have as much control over the pre-understanding as possible (Dahlberg et al., 2008; Nyström, 2012). The preliminary interpretations were further developed by using the understanding of the interactions from the field notes in order to create an understanding of what was said between the lines and to enable an explanation of the material (Ricoeur, 1976). The interpretations were further developed by being combined when more than one interpretation explained the same thing in two different ways, or rejected when the interpretation did not explain the phenomenon. This resulted in eight preliminary interpretations. An open attitude was adopted during the interpretation in order to not ascribe something that did not belong to the phenomenon (Dahlberg et al., 2008; Gadamer, 1960/2004). The interpretations were checked against the following validity criteria, applied through a movement between the parts (interpretations) and the whole (interviews) (Dahlberg et al., 2008)

- The source of an interpretation should only be an actual piece of data.
- An interpretation should not leave a considerable amount of data unexplained.
- There must be no contradictions in the same data concerning interpretations that are considered valid.

Whilst controlling the validity criteria, parts of the preliminary interpretations were developed further and parts of the preliminary interpretations were rejected because they did not fulfil the validity criteria, thus resulting in three interpretation themes. In order to create a main interpretation, the interpretation themes were reviewed against each other in order to embrace similarities. This was performed to create a constant pattern in the main interpretation and to raise the interpretation themes to a higher level of abstraction. Further, by a movement between the interpretation themes and the main interpretation they were tested against each other to fulfil validity criteria (Dahlberg et al., 2008):

- No data of relevance may be omitted in the main interpretation.
- The tentative interpretations are to be related to the main interpretation and vice versa.

### Ethical considerations

Ethical approval and permission to undertake the study were obtained from the Regional Ethical Review Board at Linköping University, Sweden (Dnr 2015/78–31). All participants received both written and verbal information and consented to participate in the study. The information given to the patients included their right to conclude their participation at any time without any risk of affecting their care, and with no further questions asked (Declaration of Helsinki, 2008). The pairs of students and supervisors were first given information by the first author and were then asked about participation by the head supervisor. The patients were first given information and then asked about participation by the nurse responsible for their care, and those who chose to participate were given further information by the first author.

## Results

The findings are presented as three interpreted themes consisting of variations of the phenomenon “learning space”, followed by the main interpretation.

### Interpreted themes

#### An interpersonal linkage based on responsibility and respect is created

The patients, the students, and the supervisors are placed in the learning space and are thus linked to each other. The interpersonal linkage that occurs within the learning space is strengthened through the taking of responsibility and the mutual respect being shown, and a balance between cooperation and independence can be promoted. Problems in cooperation occur when mutual respect is not shown, and this in turn leads to their cooperation being less effective.

The interpersonal linkage creates possibilities for a movement between the students working individually and cooperating. This is shown through respect for each other’s learning, and how their own learning affects the other. The students thus give each other the opportunity to develop independence, which is supported by them turning to each other for reflection. One student says:

We talked about this patient and that a blood test should be taken, and I felt that Maja should get to practise this on her own. She is ready for that, to do it on her own and we had time for reflection afterwards.

By listening and showing respect towards each other the students are creating supportive cooperation. On the other hand, students who do not succeed in showing respect towards each other are learning at the expense of the other; this occurs when one puts the responsibility for his/her own actions upon the other. A student says: “I have the type of personality who will easily take charge and you (student colleague) become passive. Or passive is perhaps the wrong word but you let me override you, you shouldn’t do that”. Students who act in this way are not taking responsibility for each other’s learning and have difficulties in creating supportive cooperation.

An important interpersonal linkage is created through the students’ encounters with patients, which has a potential to develop into a caring relationship depending on whether trust has been established between them. The patients have no responsibility for the students but show respect towards them by being available for them in the learning space. The difficulty within the interpersonal linkage between the patients and the students is based on the patients’ vulnerability and the difficulty patients have in protecting themselves in the learning space, due to their being in a position of dependency when being cared for. However, there is potentially genuine care made available by the students spending their time and showing respect to the patients in order to create a supportive relationship. A patient says: “It went very well, they listened to everything I said, I was neither afraid nor worried”.

In order for the students to be given the opportunity to be independent in the encounter with the patient the interpersonal linkage between the students and the supervisors becomes protracted and the supervisory control is thus reduced. This occurs when the supervisor does not participate in the encounter with the patient but is available through listening and being alert in the case of expressions of dissatisfaction or concern. There is a difficulty in being responsible at the same time as the control is reduced; one supervisor says: “It’s difficult to remain passive because you want the patient to get the help they need”. When the supervisor does not show sufficient respect in relation to the students’ requirements for learning, the movements between cooperation and independence become less effective. One student says: “I felt that the supervisor interrupted too much. I would have liked her to stay more in the background, it felt like she jumped in too quickly. You didn’t get the chance to say everything, and that’s a pity”.

#### Opportunities for support are created through interaction

The way the participants respond to each other within the learning space is significant for whether the creation of a supportive interaction is helped or hindered. A supportive interaction is characterized by mutual concern, an understanding of each other, a space where one can admit one’s own lack of knowledge and ask questions. This enables interaction with dynamic movements that are aimed at a common goal, which in turn generates an opportunity to exchange support. On the other hand, an interaction characterized by a focus directed towards one’s own needs and a lack of understanding of the rules of working together generates an interaction that lacks harmonizing movements and reduces the possibilities for a supportive exchange.

A dynamic interaction is created by students who are available for each other, help each other, and share a view of how learning should take place. This means that the students give to and receive support from each other through having created an interaction that generates security. A pair of students say: “We work well together you and I, it feels like it’s working. If I’d been by myself it would have felt a bit uncomfortable, but when you are two it feels more secure because you have someone to talk to”.

A dynamic and secure interaction between the students also enables a supportive interaction with the patient, where they focus their attention on the patient. One patient says: “Above all, I feel good when they try to make eye contact with me. Say hello, are polite and pleasant, that gives you a good first impression”. At the same time, the patient is supportive by sharing their stories so that students can learn to provide care.

When the students are not considerate and do not understand the other’s needs for learning, the interaction lacks harmony and becomes fragmented and the supportive capability is reduced. The interaction is then characterized by their being self-centred and impatient, which creates frustration. One student says:

I know it myself, I’m impatient. So taking a step back and talking about: what do you think about this? How do you want to do it? And then you think that we’ve been standing here for a while and talked and got no results. Can we do something now, and talk later when we’ve done the most necessary tests?

Interaction without harmony also occurs when the students do not succeed in being responsive towards the patient, which contributes to reduced support for the patient. A lack of harmony within the interaction contributes to a fragmented interaction, which is characterized by students not understanding the patients’ needs and leads to a patient choosing not to share his/her story. One patient says: “I feel unhappy after everything I’ve been through. It sounds odd but the cheerful attitude becomes annoying”. The interaction in these situations is characterized by the students and the patient passing each other and thus the possibility for giving and taking support is reduced.

A supervisor who is in attendance and supportive is crucial for students who themselves are unable to create a structure and routines for their interaction. A supportive interaction between the students and the supervisor is explained by the ability of the supervisor to be thoughtful and understanding towards the students’ needs, and thus a movement towards a common goal is created. For the supervisors “it’s important with a sense of togetherness, that a student learns this” and that they thus contribute to a dynamic interaction to support the students. When the interaction does not work it is due to the students and supervisors not having a common goal and structure, which causes disorientation within the learning space. The supervisor considers the students to be capable of working on their own; meanwhile the students need support in order to avoid fragmented learning. One student says: “Both he and I felt totally abandoned. We stood there and I don’t know whether it was the supervisor’s fault …”. This was particularly evident in a new relationship between students and a supervisor when the latter had a limited awareness of the students’ level of knowledge.

#### A flexible interaction within the learning space is required

Movements occur between the students, patients, and supervisors in the learning space in terms of receiving, giving, and taking place. Flexibility towards each other is needed in order for the exchange of place to occur, which is concerned with each being interested in the others’ questions, thoughts, and experiences. Possibilities for sharing experiences are created by the voluntary giving of some place through listening, observing, and giving the opportunity to take an initiative.

An exchange of place occurs when both have the opportunity to take initiatives and create an exchange of knowledge when they supervise each other in the encounter with the patient. A supervisor talks of how the students learn by exchanging knowledge:

Maja (second year student) was going to take a blood test for one patient and Anders (third year student) supported her. Anders has been supervising Maja a lot these days, Anders learns by explaining and putting his knowledge into words and provides Maja with the knowledge that she needs.

Being able to demonstrate and explain in their own time becomes a prerequisite for learning, which becomes a give-and-take situation when one student takes a step back to observe. The other student thus gets the opportunity to take the initiative. The exchange of positions between the students also occurs through a tacit agreement where there is flexibility under favourable conditions. One student says:

we almost have a silent understanding, a social understanding for when it is time to talk. I think that worked out well during the conversation, you Viktor (student colleague) could ask a follow-up question, at the same time as I tried to be quiet. I don’t think that it’s something that needs to be practised, because we met for the first time a couple of days ago. It’s something you just have, a feeling.

On the other hand, an imbalance in listening and providing space for each other’s initiatives hinders the opportunity to share experiences and knowledge. This occurs, amongst other things, when one of the students is too empathetic towards his/her student colleague’s needs and “… doesn’t dare to take too much space”.

When the students are taking place in the learning space, the patients give place to the students due to their vulnerability and dependency. The patients’ vulnerability contributes to a desire to be cared for, which leads the patients to exposing themselves and allowing the students to care for them. The patients are more or less tied within the learning space and receive care based on the students’ ability, which means that the attention the patient receives is dependent on the learning conditions. One patient says: “the students tend to take more time for their patients if they are secure, but if the students are insecure they don’t have any time at all for their patients”. On the other hand, when the students are able to care for the patient in favourable conditions a balance between giving and taking place occurs and the patient has the opportunity to share his/her experiences with the students, and the students provide security by listening and paying attention to the patient.

The supervisors’ main responsibility is for the patients’ well-being and need for care, and the students’ space is dependent on these needs. For example, in emergency situations students have to take a step back whether they want to or not. On the other hand, the supervisors try to balance the give and take of space based on students’ learning requirements by listening to them. A favourable learning space is created when the supervisor takes a step back and provides space and the opportunity to take initiatives. One supervisor says:

I try to be laid-back but I don’t always succeed, it’s really difficult to remain silent, and stand with my hands on my back, it’s not really my way of doing things in ordinary life, so it’s difficult to do it as a supervisor.

### Main interpretation

An interpersonal linkage between the patient, the students, and the supervisor provides space for interpersonal movements. An ability to show respect and to take responsibility balances these movements and creates reversibility between cooperation and independence. A potential learning space based on reciprocity is thus created. When respect is not shown within the learning space then the linkage becomes difficult, thus affecting the possibilities for a well-functioning relationship. The latter is characterized by parallel actions with a low degree of interaction, which consist of unbalanced movements between independence and cooperation. The interpersonal movements are more or less dynamic and are dependent on the students’ and supervisors’ awareness of the others and their ability to take responsibility within the learning space.

A learning space based on mutual respect creates the prerequisites for beneficial and supportive interactions that contribute to a deeper relationship. Dynamic movements are essential in creating supportive cooperation. When the movements are dynamic and directed towards a common goal, the cooperation is characterized by reciprocity, transparency, and understanding. The prerequisites for giving and receiving support within the learning space are created through openly declared or tacit agreements. When the movements diverge, the focus is directed towards one’s own needs, thus making it more difficult to pay attention to the others’ need for support. Furthermore, the consequences of a less dynamic cooperation create a learning that is elusive due to its lacking coherence. A supportive cooperation is, on the other hand, based on interpersonal relationships with dynamic movements, which strengthens the interpersonal linkages between patient, students, and supervisor and contributes to genuine care and learning.

The supportive and dynamic cooperation between the patient, the students, and the supervisor deepens their relationship, and the necessary conditions are provided for observing each other’s needs for giving and taking of some place within the learning space. A favourable learning space is thus constituted of movements between getting, giving, and taking some place as a prerequisite for optimal learning. A varying degree of reversibility between getting, giving, and taking place is dependent on the extent to which there is an adherence to the interpersonal movements. A mutual exchange is developed when place is provided for enabling initiatives and for utilizing each other’s knowledge and experience. Difficulties occur when attention is given involuntarily within the learning space and when initiatives are taken without compliance. A flexible movement between giving attention and taking initiatives creates possibilities for strengthening the interpersonal connections between the patient, the students, and the supervisor. The learning space thus has the potential for creating both dynamic interpersonal movements that generate development, and interpersonal movements that get out of step and generate an involuntary bonding that is not balanced.

A reciprocity between the patient, the students, and the supervisor demands that the balance is monitored. An imbalance between the parties in the learning space demands that support and attention are given in order to maintain a balance that can enable a reciprocal exchange. If the learning space is not given high priority, even for a single moment, then there is a risk of insecurity and imbalance within the interpersonal movements, and the learning is likely to be fragmented.

Due to the inevitable interpersonal connections between the parties and the fact that the patients are the hub of the learning space, they find themselves in a position of dependency where they are unable to defend themselves from the learning space. In addition, when there are no dynamic movements there is a risk that the parties become quiet, leading to the learning space diminishing and becoming limited and less accessible. The balance between the parties must be paid attention to and monitored in order for favourable learning to occur.

## Discussion

### Of method

This study has been carried out in accordance with reflective lifeworld research (RLR), with a hermeneutic approach. This approach was chosen in order to enable an explanation of the learning space, which has been shown in previous studies to be created in the encounter between patients, students, and supervisors. One specific methodological challenge in terms of validity and objectivity was the balance between using pre-understanding according to the hermeneutic approach and at the same time bringing it in line with the RLR approach (Dahlberg et al., 2008). Analysing with an interpretive approach allowed the use of pre-understanding in order to create an understanding of what was said between the lines and to enable an explanation of the phenomenon (Ricoeur, 1976), which in this study was the learning space. In the other part of the analysis the pre-understanding should be bridled, in order to control the interpretations supported by the validity criteria (Dahlberg et al., 2008). The challenge entailed balancing between using and bridling the pre-understanding where the validity criteria were helpful and constituted a guide in the reflective process of analysis.

The interviews lasted for approximately 10 to 20 minutes; the shorter interviews were exclusively with the patients, due to their being tired and not pressurized to answer any questions. In view of the brevity of the interviews with the patients the observations are particularly important for creating an understanding of the patients’ experiences of the learning space. This is due to the interview questions being based on the observations of the interpersonal interactions and thus solely focused on the phenomenon “the learning space”. Due to the abstract nature of the phenomenon, the interview questions could thus not be specifically asked in terms of the “the learning space”. The questions related to the learning space were thus formulated as: “How did you experience the encounter when the students were caring for you?” The interviews and the observations created a total understanding of the patient experiences. Despite these difficulties, rich descriptions of the learning space were revealed during the analysis and enabled interpreted themes and the main interpretation. Through the abstract level of the understanding of the phenomenon in the main interpretation, transferability to other similar contexts for nursing students’ clinical practice can be implemented. This is in line with van Wijngaarden, van der Meide & Dahlberg (2017), who emphasize that validity is strengthened by the main emphasis in research, which is not what the informant says, but the meaning structure of the phenomenon.

### Of results

This study shows the importance of a favourable learning space for enabling students to learn to care in clinical practice. The learning space exists in a caring context, where learning and caring interact, which entails the students learning while they care for the patient. The learning space consists of interpersonal interactions between the students, the patients, and the supervisors. Depending on the parties’ ability to show respect towards each other and to take responsibility, the interpersonal interactions become more or less dynamic. These interpersonal movements between the students, the patients, and the supervisors are a prerequisite for creating well-functioning and supportive relationships within the learning space. In the following discussion, the dimensions of the interpersonal relationships, dynamic movements, and opportunities for support will be highlighted.

Well-functioning relationships between the patients, the students, and the supervisors within the learning space are based on respect being shown towards each other, and students and supervisors taking responsibility for the patients’ care. The quality of the interactions between students in pairs and the support from supervisors is thus important when caring for patients. This is in line with earlier research showing that the creation of favourable relationships in clinical practice is important while learning to care. Previous research about relationships within clinical practice has focused on students who learn together (e.g., Stenberg & Carlson, 2015), students and the patients they care for (e.g., Suikkala, Leino-Kilpi, & Katajisto, 2009), and students and their supervisors (e.g., Nygren & Carlsson, 2017). This study makes further contributions towards an overall perspective by presenting the learning space, where the focus is directed towards relationships that involve three perspectives: from the patients, the students, and the supervisors.

The results of this study show that unfavourable relationships created within the learning space are based on a lack of ability to show mutual respect. This in turn affects the cooperation between the students, the patients, and the supervisors, which thus becomes less effective and characterized by a low degree of favourable interaction. Previous research, from both a student and a patient perspective, has shown that a mechanistic relationship is characterized by patients being passive and quietly observing the students while the latter practise technical skills (Suikkala & Leino-Kilpi, 2005). In addition, our results show the importance of creating a sense of trust between the students and the patients in order to enable a supportive relationship. This concurs with the findings of Eskilsson et al. (2015), who showed that the patients are sometimes doubtful about the students’ ability to care for them and whether the students receive sufficient support from their supervisors in order to “shoulder” the responsibilities of caring for the patients. Similarly, Sandvik, Eriksson, and Hilli (2015) describe the relationship between the students and their supervisors as being important for creating feelings of safety and security in students’ learning. Regardless of whether the relationship between a student and a patient or between a pair of students and a patient is unfavourable or not, the learning space requires both the presence and the accessibility of the supervisors, in order to be supportive. Our results provide a deeper understanding of the importance of the relationships, due to the focus on the whole, which includes all the perspectives within the learning space. This can be seen in the complexity that exists in the learning space where all relationships, both favourable and unfavourable, constantly affect each other. The learning space thus needs to be balanced in terms of the creation of beneficial interpersonal interactions as well as being understood as a whole.

Another aspect of the results is the importance of respect being shown towards each other in spite of the complexity of the different positions, situations, roles, and aims that the three parties have in the learning space. This complexity includes the students being dependent on support from their supervisors, the patients being dependent on the relationship with students and supervisors, and the supervisors being responsible for supporting students and patients. Our results show that due to this complexity, the parties need to show respect towards each other in order to create a favourable learning space. This concurs with previous research showing that the students’ meetings with the patients are seen to be the basis for students’ learning (Manninen et al., 2012). Eskilsson et al. (2015) also emphasize that a mutual trust between the supervisors and the students is provided when the supervisor is able to remain in the background while students are caring for the patients. However, the relationship between the students and the patients has been shown to be balanced by the supervisor, who attempts to create both favourable learning and caring (Eskilsson et al., 2015; Manninen, Welin Henriksson, Scheja, & Silén, 2015). In addition, our results show that a lack of ability in showing respect towards each other within the learning space reduces the possibilities for creating interpersonal relationships, while the cooperation between the three parties also becomes less effective. Furthermore, our results also show that well-functioning relationships within the learning space create opportunities for the pairs of students to care for the patients independently, while being supported by their supervisor.

Another feature of the results is the more or less dynamic movements in interpersonal interactions, which affect how well-functioning the relationships within the learning space become. The well-functioning relationships enable dynamic movements, which are aimed at a common goal. On the other hand, a lack of ability in creating a favourable relationship is based on diverging movements, where the focus is directed towards one’s own needs instead of a focus on each other’s needs within the learning space. We emphasize that dynamic movements are promoted by a reflective approach, in order to create an awareness of the self in relation to the experienced situation, the others, and their needs. This is in line with Ekebergh (2007), who maintains that reflection can support learning by the individual becoming aware of him/herself in relation to the experiences and to developing a greater understanding of the experienced situation. Learning by reflection has also been shown, from a student perspective, to support their learning in understanding their own emotions and in self-assessment (Fernandez-Pena et al., 2016; Sandvik et al., 2015).

Furthermore, our results show that the movements within the learning space are also described in terms of receiving, giving, and taking place between the students, the patients, and the supervisors. A balance between receiving, giving, and taking place enables the students to give place to each other, by one student taking a step back to observe, enabling the other to take an initiative. This is supported by Holst and Hörberg (2012), who showed that students when learning together were able to support and learn from each other by providing space for each other. In addition, our results show that students who have a balanced cooperation are able to create a good caring relationship with the patients based on providing place for the patients, by listening to their stories. The importance of listening to the patients’ stories in order to create a caring relationship between the students and the patients has also been emphasized by Ekebergh (2009) and Gidman (2013). On the other hand, our results also show that students who are unable to be responsive towards the patients contribute to reduced support for the patient. This concurs with Eskilsson et al. (2015), who emphasize in line with our results that the patients are dependent on the students’ ability to provide care. This is shown in our results in terms of the patient being vulnerable and dependent, which contributes to a desire to be cared for. Patients’ vulnerability is also highlighted by Todres, Galvin, and Dahlberg (2014), who maintain that a lifeworld-led approach can respond to the patients’ needs for recognition by focusing on the common humanity between the carers and the patient in the shared moment. This demonstrates the importance of both students and supervisors having a lifeworld-led perspective (Dahlberg, Todres, & Galvin, 2009) in the learning space, because patients are potentially vulnerable when they are being cared for by students.

We believe that the ability to create dynamic movements that enable a movement between receiving, giving, and taking place is dependent on the capacity for reflection among students and their supervisors, which can enable them to create an awareness of the situation as a whole. In addition, a reflective and lifeworld-led approach in caring enables the students and the supervisors to create an understanding of the patients’ need for support within the learning space. This is in line with Ekebergh (2007), who describes lifeworld-based learning and caring as gaining a greater understanding of reflection in relation to human consciousness.

The third dimension to be highlighted concerns opportunities for support within the interpersonal relationships in the learning space. Our results show that the way the patients, the students, and the supervisors respond to each other is significant for whether a supportive interaction is created or not. This is in line with Stockhausen (2009), who from a patient perspective shows that they strive to support the students’ learning by being available for being cared for and by encouraging the students. In addition, our results show that supportive relationships within the learning space are further characterized by mutual concern, an understanding of each other, and a space where one can admit one’s own lack of knowledge and ask questions. This in line with Suikkala and Leino-Kilpi (2005), who stated that students with an understanding of their patients’ welfare provided comfort and encouragement to the patients. Moreover, our results show that interpersonal interactions where the focus is on one’s own needs generate a lack of harmonizing movements, thus reducing possibilities for supportive exchange. More specifically, our results show that lack of cooperation between the students contributes to reduced support for the patients, which creates a need for support from the supervisors in order to create structure and routines for the interactions within the learning space. This concurs with Nygren and Carlson (2017), who emphasize from a supervising perspective that an imbalance between students in peer learning required the supervisor to support the students in order to balance the students’ cooperation and individual development. Sandvik et al. (2015), from a students’ perspective, also demonstrate that the supervisors provide students with support by balancing the degree of responsibility they give to the students. In addition, our results show that the learning space is supported by supervisors being thoughtful and understanding towards the students’ needs, which enables dynamic movement towards a common goal. Furthermore, responding to each other by showing interest in each other’s contributions to the learning space, the patients, the students, and the supervisors can develop an understanding of each other´s situation and thus also support each other. We maintain that the learning space has a potential for being supportive, which is based on the ability of the parties to be in attendance with mutual concern towards each other despite the possibility of unfavourable cooperation with a low degree of support being created. Moreover, the ability to provide support should also be understood as constantly changing depending on the situations that arise within the learning space. The patients, the students, and the supervisors should thus always be observant and attentive towards each other’s need for support.

### Concluding reflections

In conclusion, the aim of this study was to explain and understand the learning space that occurs in the interaction between patients, pairs of students, and supervisors. The phenomenon in this study is complex, since it includes the three perspectives; however, we are aware that this could be further compounded through the existence of additional actors, such as other nursing staff and professionals, or the patients’ relatives. The phenomenon is also further complicated by its being in constant change, which entails the parties entering into and leaving the learning space. This could explain the expandable nature of the learning space and its not being isolated from the surrounding environment. The learning space is affected by how the patients, the students, and the supervisors respond to each other. Depending on the degree of respect, well-functioning relationships, and interpersonal dynamic movements, a more or less supportive interaction is created within the learning space. This should also be understood vice versa, as the level of mutual support is important for the development of well-functioning relationships and interpersonal dynamic movements. Furthermore, in order to exploit the potential of the learning space it is of importance to understand and consider the learning space as a whole including the patients, the students, and the supervisors. This is particularly the case when clinic and academia are collaborating and planning nursing students’ clinical practice.
